# The effects of a low-energy, high frequency liquid optic interface femtosecond laser system on lens capsulotomy

**DOI:** 10.1038/srep24352

**Published:** 2016-04-19

**Authors:** Geraint P. Williams, Ben L. George, Yoke R. Wong, Xin-Yi Seah, Heng-Pei Ang, Mun Kitt A. Loke, Shian Chao Tay, Jod S. Mehta

**Affiliations:** 1 Tissue Engineering and Stem Cell Group, Singapore Eye Research Institute, Singapore, Singapore; 2 Corneal and External Eye Disease Service, Singapore National Eye Centre, Singapore, Singapore; 3 Biomechanics Laboratory, Singapore General Hospital, Singapore, Singapore; 4 Department of Hand Surgery, Singapore General Hospital, Singapore, Singapore

## Abstract

The introduction of femtosecond laser assisted cataract surgery (FLACS) is a paradigm changing approach in cataract surgery, the most commonly performed surgical procedure. FLACS has the potential to optimize the creation of an anterior lens capsulotomy, a critical step in accessing the cataractous lens. The merits of using a laser instead of a manual approach include a potentially more circular, consistent, and stronger aperture. In this study we demonstrated for the first time in both a porcine and human experimental setting that with a low energy, high repetition FLACS system, that a circular, smooth and strong capsulotomy was achievable. While there was no demonstrable difference in the resistance to rupture before or after the removal of the nucleus, larger capsulotomies had an increase in tensile strength. The LDV Z8 system appeared to create circular, rupture-resistant and smooth capsulotomies in both porcine and more importantly human globes.

Half of the worldwide burden of blindness, standing at 39 million in 2012, is accounted for by cataract with large-scale population based studies showing an incidence of up to 36% in older populations^1^,^2^,^3^. Unsurprisingly, cataract surgery is one of the most commonly performed surgical interventions worldwide, with 15 million procedures performed per annum globally^2^,^3^,^4^. This is conventionally achieved through small-incision surgery combined with phacoemulsification and removal of lens material prior to insertion of an intra-ocular lens (IOL). In order to facilitate cataract removal and IOL implantation, an opening in the anterior capsule is achieved by a free hand technique with the assistance of surgical instruments, termed curvelinear continuous capsulorhexis (CCC)^5^.

Femtosecond Laser Assisted Cataract Surgery (FLACS) has been heralded as a paradigm shift in undertaking cataract surgery by automating key steps in the surgical procedure^6^,^7^,^8^,^9^. In particular the advent of femtosecond laser technology offers a potentially more consistent and reliable means of achieving the anterior lens capsule opening, re-named capsulotomy. This may ‘reduce and flatten the learning curve’ in one of the critical steps during surgery for trainees^7^.

Considerations in judging the success of capsulotomy include consistency in size, position, circularity and strength. FLACS has been advocated because of an improvement in the circularity of the capsulotomy compared to manual capsulorhexis, greater predictability in achieving the desired size and centration of capsulotomy and in turn effective lens position (ELP)^10^,^11^. ELP has implications for success of multi-focal or toric lens implantation used to overcome astigmatism, where movement or poor centration may adversely affect the quality of vision^6^,^12^,^13^. The ability to correct astigmatic refractive error at the time of cataract surgery through toric intra-ocular lenses may become more commonplace and with a prevalence of >25% among Europeans, may precipitate a drive towards this technology^14^.

Although consistent circularity and predictability in size have been demonstrated with the Catalys, Victus, LenSX and LensAR systems^13^,^15^,^16^,^17^,^18^, the advent of femtosecond laser derived capsulotomies has been associated with capsular tags. Concern over tags has been raised with FLACS not least as they may be complicated further by anterior capsular tears^19^. This is important as IOLs are typically positioned in the lens bag by folding or injecting and must therefore resist forces that could lead to rupture. The morphology of FLACS capsulotomy edges, characterized by a more serrated appearance compared to manual capsulorhexis when viewed at high magnification (>×2000) may play a role in vulnerability to tags and tears^20^,^21^,^22^. It is perhaps surprising therefore that the existing literature suggest that many of the femtosecond laser platforms compare favorably to, but also exhibit high mechanical strength compared to manual rhexis (Table 1)^13^,^15^,^16^,^17^,^18^.

Judging mechanical strength across platforms and extrapolating human relevance is challenging because most studies are carried out in porcine systems, by myriad of approaches including evaluation of strength *in situ*, with or without lens nucleus removal and with different instrumentation (Table 1)^10^,^12^,^15^,^16^,^22^,^23^. The Ziemer LDV Z8, which uses a liquid optic interface to facilitate laser capsulotomy and lens fragmentation, in contrast to most systems, is a low energy high frequency system^9^,^24^,^25^. The merits of the system are that it operates in the nanojoule range and we have previously shown that it elicits minimal inflammatory response and apoptosis in laser refractive procedures^26^,^27^.

The aim of this study therefore was to determine the effects of the Ziemer LDV Z8 liquid interface femtosecond laser platform during capsulotomy for a range of parameters including circularity, elasticity, predictability of size, edge morphology and mechanical strength with the nucleus intact and following removal in both porcine and human eyes.

## Results

Porcine capsulotomy evaluation.

### Capsule size and circularity

**Figure 1:**
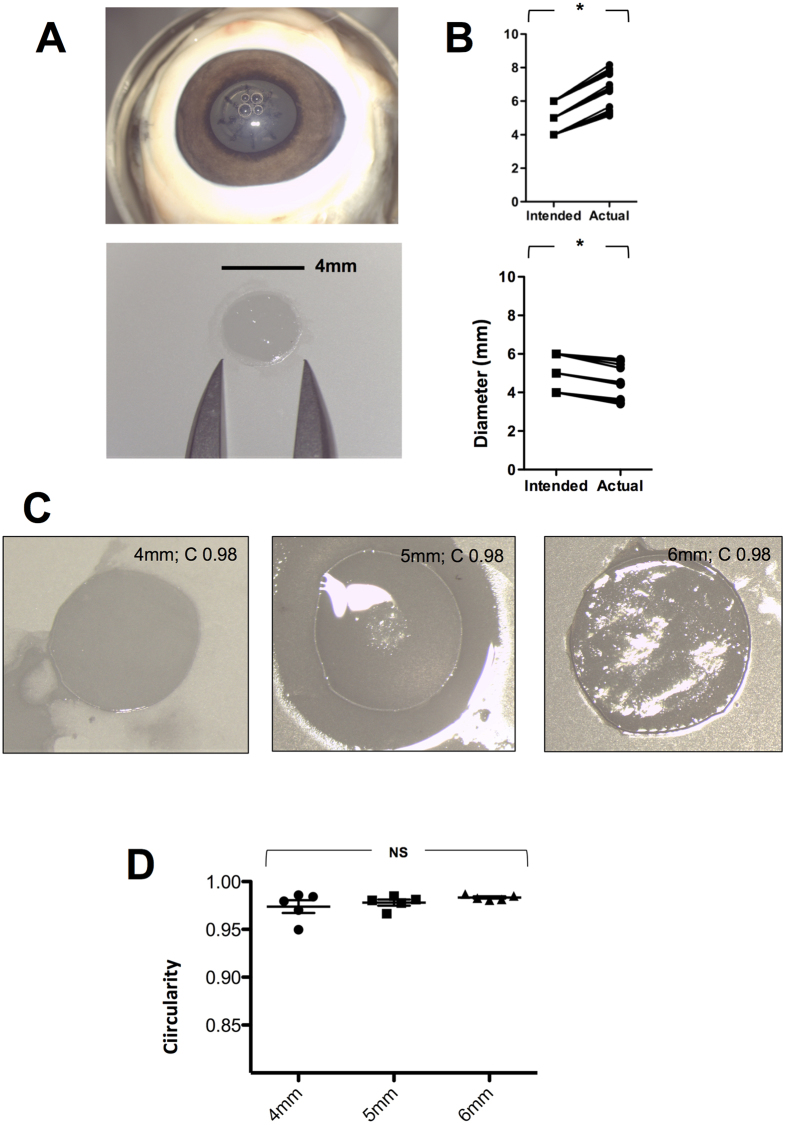
Capsule retrieval, time to completion and circularity. Color photographs showing the capsulotomy and fragmentation pattern *in situ*, retrieval, measurement and mounting of lens capsule prior to preparation for scanning electron microscopy (**A**). The intended and actual size of capsulotomy (upper panel) and capsule (lower panel) (**B**). Representative photographs of lens capsule circularity at 4, 5 and 6 mm (n = 18) (**C**) and showing comparison at different sizes (n = 15) (**D**). **p < 0.01, NS = Not Significant

All procedures were completed successfully without complication. A consistently larger capsulotomy size than programmed (4 mm vs. 5.3 mm, 5 mm vs. 6.7 mm, 6 mm vs. 7.7 mm; p < 0.05) was observed (n = 18) (Fig. 1A,B). A commensurate shrinkage size in the capsule was observed (4 mm vs. 3.5 mm, 5 mm vs. 4.45 mm, 6 mm vs. 5.6 mm; n = 18 p < 0.05), reflecting porcine lens elasticity. The lens capsule circularity achieved was 0.98 [n = 15; range 0.95–0.99] with no demonstrable influence of size with larger capsulotomy (p = 0.23) (Fig. 1C,D).

### Capsule edge

**Figure 2:**
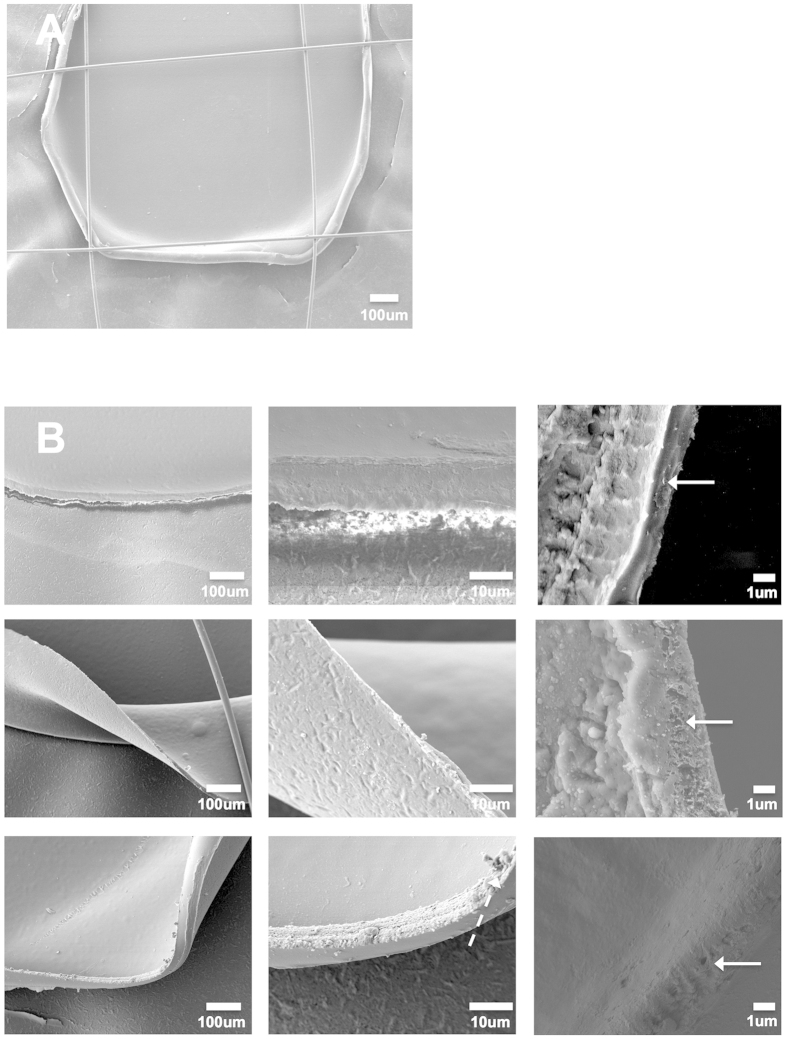
Capsule edge morphology at different magnification with the Z8 femtosecond laser capsulotomy. Scanning electron microscopy (SEM) images of capsule edge taken at low magnification (**A**) ×140, ×750 and ×4000 magnification for 4, 5 and 6 mm capsulotomy (n = 18) ((**B**) upper to lower rows). A micro-tag is highlighted with a hatched arrow and the capsule edges at high magnifications by straight arrows.

A representative low magnification image of the membrane-mounted capsule is shown with overlying sutures (n = 18) (Fig. 2A). Removable non-significant micro-tags were evident on SEM in 2/18 cases but otherwise relatively smooth edges were observed and no anterior capsule tears occurred (Fig. 2B).

### Capsulotomy strength

**Figure 3:**
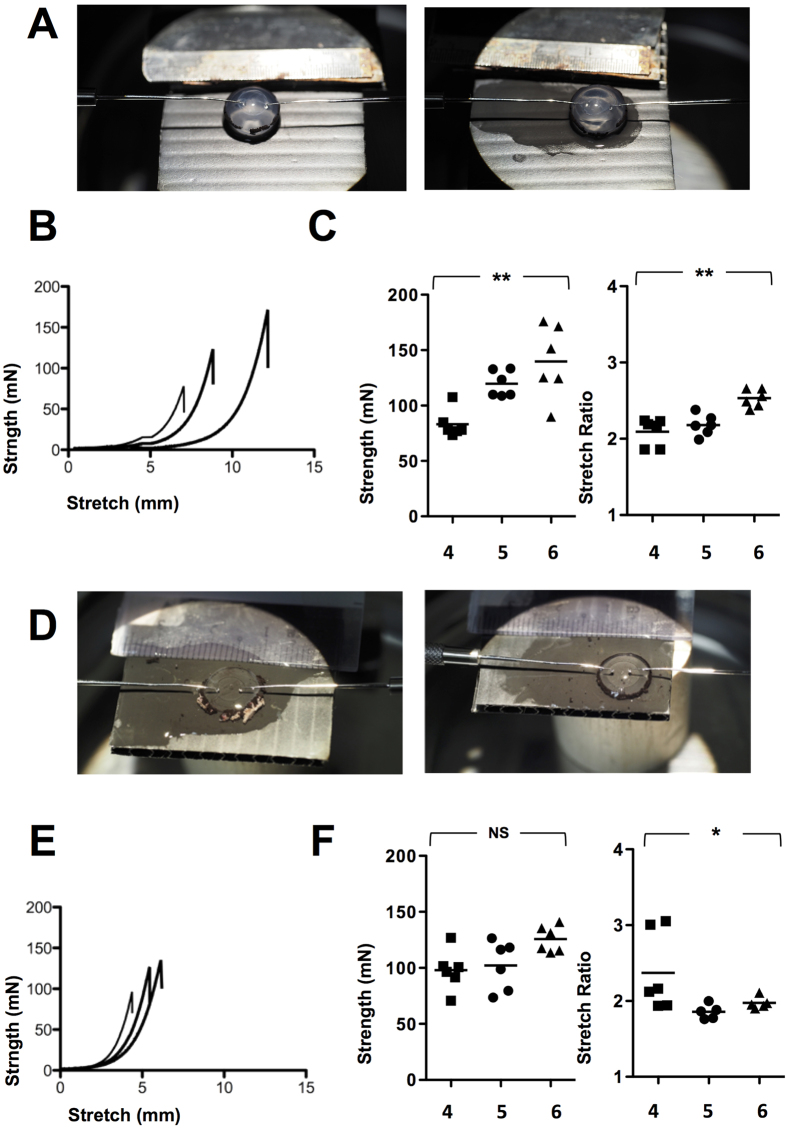
Lens capsule strength at 4, 5 and 6 mm capsulotomy with and without nucleus. Lenses were removed with nucleus intact and resistance of the capsulotomy to rupture was measured in mN and the stretching ratio by (size mm + displacement mm)/size. Two mushroom shaped pins were placed posterior to the capsulotomy edge and the rate of pin displacement was set at 6 mm/min (**A**). Comparison between strength and stretch ratio at 4, 5 and 6 mm are shown in in (**B**,**C**) (n = 18). Experiments were repeated following removal of nucleus (**D–F**) (n = 18) *p < 0.05 and **p < 0.01.

There was an incremental increase in capsule tension strength from 4 to 5 to 6 mm capsulotomy with intact nucleus, measured at a median of 78.1 mN[range 73.4–107.7] vs. 116.7[108.9–133.5] vs. 138.3[89.9–175.9] (n = 18); <0.001) with a 4 mm capsulotomy showing significant reduction in resistance to rupture compared to 5 or 6 mm openings (Fig. 3A–C). A higher stretch ratio was observed with a larger capsulotomy, measured at 2.2[1.9–2.24] vs. 2.2[2.0–2.4] vs. 2.5[2.4–2.6] in the 4, 5 and 6 mm groups (n = 18); <0.01).

A corresponding trend was also observed in strength following removal of the nucleus, with a rupture force of 98.0[70.8–126.9] vs. 107.5[73.5–126.5] vs. 124.3[113.6–141.0] although this was not significant (p = 0.06). There was no consistent pattern in stretch ratios following nucleus removal for 4 mm (2.2[1.9–2.2] vs. 5 mm (1.9[1.8–2.0]) vs. 6 mm (2.0[1.9–2.1]) (n = 18); p < 0.05) with a significant difference between a 4 and 5 mm capsulotomy (Fig. 3D–F).

There was no difference in strength at each capsulotomy size with the nucleus *in situ* versus removal (n = 12); p = NS at 4, 5 and 6 mm capsulotomy respectively). A reduction in the stretching ratio was observed at 6 mm capsulotomy following nucleus removal however 2.5 vs. 1.9 (n = 12); p < 0.01).

### Human capsulotomy evaluation

**Figure 4:**
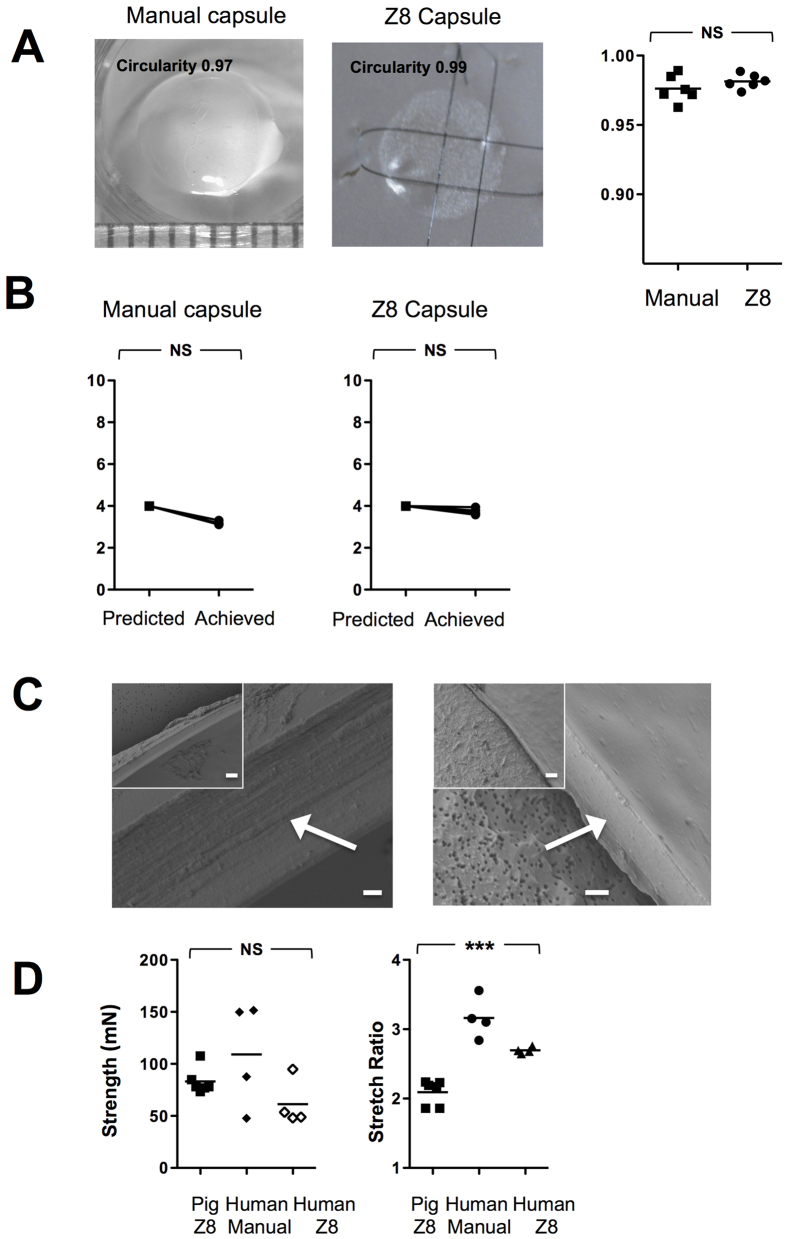
Human lens capsule circularity, edge and strength. Human lenses were removed with nucleus intact and circularity (**A**) and elasticity (**B**) of the capsule were evaluated (n = 12). Scanning electron microscopy (SEM) images of capsule edge by manual and Z8 created approach (**C**). The inset image is at ×750 magnification (line denotes 10 um) and the larger image at ×4000 (line denotes 1 um) and the capsule edge is arrowed. Resistance of the capsulotomy to rupture was measured in mN and the stretching ratio by (size mm + displacement mm)/size. Two mushroom shaped pins were placed posterior to the capsulotomy edge and the rate of pin displacement was set at 6 mm/min. Comparison between strength and stretch ratio by porcine Z8, human manual or human Z8 are shown in (**D**) (n = 14). **p < 0.01. NS = Not Significant.

Median circularity 0.97[0.96–0.99] (n = 6) was comparable to the Z8 0.98[0.97–0.99] (n = 6) (p = 0.3) but with a slight increase in variability with manual cuts (Fig. 4A). Manual accuracy was also less predictable than with the Z8, with capsules measured at 3.17 mm[3.12–3.31] (p = 0.25) and 3.72 mm[3.58–3.94] (p = 0.05) representing a deviation from intended 4 mm size approximating 0.8 mm with manual and 0.25 mm with the Z8.

The edge of the human manual capsule showed a smooth profile with no induration or tags by both approaches (n = 12) (Fig. 4C).

The strength of the human manual at 4 mm was measured at 118.8 mN[47.8–151.5], and the Z8 cut at 51.2[48.2–94.9] but the greater variability meant this was not significantly different and overall no difference was seen with the comparable sized porcine capsulotomies ability to resist rupture, 78.1 mN[range 73.4–107.7] (n = 14); p = 0.3).

Stretch ratios were measured at 2.2[1.9–2.2], 3.1[2.8–3.6] and 2.7[2.7–2.8] (n = 14; p < 0.001) for the porcine, human manual and human Z8 capsulotomies respectively. This represented a significant difference with post hoc testing revealing a higher stretch ratio for the human lens capsulotomies than the porcine, the human manual compared to human Z8 but with the greatest effect in a manual capsulorhexis.

## Discussion

FLACS presents a paradigm shift in undertaking cataract surgery. The advent of many different and alternative FLACS platforms, predominantly operating in the micro joule energy range, has led to conflicting data regarding the efficacy of capsulotomy formation and utility^9^. To our knowledge this is the first study to offer a comprehensive evaluation of capsulotomy size, circularity, edge and strength under differing conditions with a low energy, high repetition FLACS platform.

We have shown that in porcine lenses with high elasticity, that recoil results in an enhancement of the capsulotomy size and corresponding contraction in the capsule. This effect was abrogated in human lenses with a reduction in the degree of deviation from intended capsulotomy size. Nagy and colleagues showed that for an intended 5.00 mm capsulorhexis in porcine eyes, the mean achieved diameter was 5.02 +/−0.04 mm using the LenSX femtosecond laser, and Auffarth *et al.* demonstrated a mean achieved diameter of 5.50 +/−0.12 mm (intended 5.5 mm) with the Victus platform, indicating high accuracy and predictability combined with a low variability^6^,^15^. The 0.5 mm difference in capsulotomy in our study with porcine lens capsules coincided with a corresponding contraction of the capsule to the same extent. This suggests therefore there is greater propensity for the lens capsule to stretch and behave in a fashion similar to the infant lens in humans^28^. Indeed, a recognition of the relatively inelastic state of the human lens capsule comparatively, exaggerated with age^29^, was reflected in the actual size of the femtosecond laser cut human capsules in human globes (median age 61 years), which were demonstrably closer to the intended size at approximately 0.25 mm contraction. We also showed that lens circularity was high in Z8 cut porcine and human lenses, with no variation in circularity with increased capsulotomy size. Even though circularity was good in the manual performed cases (performed by an experienced phaco surgeon having done more than 750 cases) there was less variation with the laser.

The edges of the femtosecond laser cut capsulotomy were smooth and consistent across sizes in both the porcine and human models. Some micro-tags were seen with FLACS generated capsulotomies but these were removed manually with no adverse effects^19^,^30^. Earlier literature with higher energy FLACS platforms suggested a higher rate of anterior tags and the reporting of adherence of the capsule to the underlying lens material. These changes have been supported with evidence of collagen melting and denaturation at higher energy settings^31^. The rate of anterior capsule tears however has been a source of some controversy with rates varying from 0.1% (Catalys platform) to 1.8% (Technolas), 1.84% (Catalys), 5.3% (Lensar)^20^,^32^,^33^,^34^,^35^. It is not clear whether this effect is from differing platforms alone as surgical experience appears to play role in reducing these events^19^.

The influence of edge smoothness on anterior capsular tears is still unresolved. Bala and colleagues undertook an inter-platform comparison and found that while the edges were approaching manual smoothness, there was variation among platforms with those employing soft fit interfaces (LenSX) compared to liquid/hybrid liquid interfaces^30^. This does not explain the discrepancy in the clinical data seen in the human series by Day and Abell^20^,^34^, serrated edges demonstrated in other studies with the Victus or the relatively smooth edges demonstrated in this study with the LDV Z8. The energy differential between the Victus, employing a curved face interface with ocular surface bathed in saline; Catalys using a liquid interface, (both operating in the micro joule range) and the LDV Z8 (liquid interface operating in the nanojoule energy range) has been previously discussed and needs to be considered in the context of edge creation^9^. It is possible that the edge configuration achieved with the LDV Z8 generates a smoother alignment secondary to lower energy delivery and a circular cutting motion.

The full implications of edge smoothness for resistance to rupture have yet to be established. The existing literature suggests that that the rupture force needed to break the capsulotomy/CCC is in the region of 110–175 mN by femtosecond and 65–155 mN by manual approaches^13^,^15^,^16^,^17^,^18^. There is inherent difficulty however in comparing and interpreting this data due to the different approaches taken to calculate rupture values (including the removal or leaving lens nucleus *in situ*), the removal of the capsule from the globe or cutting the edges of the capsule prior to mechanical testing. Furthermore there is variation in the size of the pins employed, the rate at which they are displaced and the machine utilized to rupture^13^,^15^,^16^,^17^,^18^.

The stretch values also can be calculated by different formulation and for this reason we adopted an approach to consistently determine the values with/without removal of lens and following extraction from the globe in both porcine and human globes. This approach, perhaps surprisingly gave consistent values with or without a nucleus. We also showed similar capsulotomy strength with other platforms reported in the literature, but with enhanced strength at an increased diameter. This is in keeping with the findings in a recent study by Packer and colleagues who showed a similar enhancement of resistance to rupture as the capsulotomy aperture is enlarged^10^. This has clear implications when considering the minimal safe diameter, not only because a consistent albeit small capsulotomy is achievable, but also that this may come at the expense of strength – both during phacoemulsification and during lens insertion. The latter may of course have a greater bearing on lenses that require additional manipulation for refractive outcomes such as toric IOLS. Although there was no effect from the removal of the lens nucleus on incremental resistance to rupture the corresponding increase in stretch ratio when the nucleus was intact was lost on nuclear removal, possibly representing the loss of support afforded by the nucleus or indeed facilitating greater compliance.

The median resistance to rupture appeared more variable in the manual group and intriguingly the elastic effects of the porcine lens appear to be negated with laser capsulotomy as the ratio was lower compared to a manual rhexis. Direct comparisons with relatively stronger 5 mm and 6 mm in manual rhexis and Z8 porcine capsulorhexis were precluded due to the poor dilatation of the cadaveric human lenses, preventing larger capsulotomies. This of course is a consideration in patients since a small pupil is a relative contra-indication to the use of FLACS. A limitation in this study is the inability to harvest cadaveric globes at a time frame commensurate with our porcine studies. Nonetheless and despite the requirement to complete lamellar dissection for the corneas to circumvent corneal edema, the strength of the 4 mm cuts were not significantly lower than for fresh porcine eyes despite the laser being fired through an optically less clear interface. Consistency and the effects of increasing energy to circumvent these problems is the subject of ongoing investigation.

Despite striking inequalities in access to cataract surgery in developing nations, increasing industrialization combined with an ageing population will present ever-increasing demands on health care resources^2^,^3^. The relative high cost of acquiring femtosecond platforms currently makes this a less cost-effective technology^36^. Second eye surgery (despite functional vision often being achieved by unilateral first eye surgery) is usually undertaken in developed countries on the grounds of addressing anisometropia (inequality in refractive outcome) and achieving stereopsis but also appears cost-effective^37^. FLACS therefore, despite its current high costs due to a relatively low uptake, could potentially increase surgical workflow by facilitating the automation of key steps such as capsulotomy in systems committed to routinely undertaking bilateral surgery. In particular technicians or even robots could theoretically increase throughput by saving surgical time for crucial steps during the procedure by a trained surgeon.

In conclusion, we have demonstrated that the LDV Z8 low energy, high repetition femtosecond laser is able to create a consistent, circular and smooth capsulotomy through a clear cornea. A reduction in the stretching ratio compared to human manual was observed albeit without compromise to strength with a small capsulorhexis capsulotomy. The laser cut capsulotomy however was capable of resisting rupture and stress at increasing diameter.

## Methods

*Ex vivo* cadaveric porcine eyes were sourced from a local abattoir, harvested at <6 post hours enucleation and evaluated with the Ziemer LDV Z8 femtosecond laser (software version ×5054) for different capsulotomy sizes (4, 5 or 6 mm diameter, 0.8 mm height, cut speed 50 mm/s, energy 90–150%) as previously described (n = 18)^25^. An additional group of eyes was used for tension experiments following removal of nucleus, n = 18 Briefly, the corneal epithelium was debrided and the globe mounted in a suction stand prior docking with a liquid patient interface followed by suction, liquid immersion and attachment of the laser head.

Human globes were acquired from Lions Eye Institue (Tampa, Fl, USA) and Sightlife (Seattle, WA, USA) following respective institutional review board approval, in accordance with approved guidelines. Consent was taken at the time of retrieval by next of kin for use for research and experimental protocols were authorised by the Lions Eye Institue (Tampa, Fl, USA) and Sightlife (Seattle, WA, USA) as human tissue was deemed unsuitable for transplantation. The median age was 61 years [range 53–70] with a median time from death to surgery of 4 days [range 4–8 days] (n = 12). Manual CCC was achieved with a 27-g needle with Viscoat ophthalmic viscoelastic device (Alcon, USA). There was no significant difference between the ages or time to procedure in the manual or Z8 groups (p = 0.82; p = 1.0). In order to circumvent the effects of corneal edema for FLACS due to the age of the donors, a manual anterior lamellar dissection to approximately 200 μm was undertaken to optimize clarity.

Lens capsules were removed and immediately placed on to a Millipore millicell culture inset, photographed (×10 magnification with a Leica microscope) and anchored to the underlying membrane with 10-0 Ethicon overlay sutures (Fig. 1A). Image J was used to determine capsule/capsulotomy size by pixilation compared to a reference rule and capsule circularity with the formula 4π×Area/(perimeter)^2^ (value of 1.0 represented a perfect sphere)^38^.

Capsules were washed twice in 1× Phosphate Buffered Saline (PBS) for 10 minutes each before being immersed in 1% aqueous solution of osmium tetraoxide (FMB, Singapore) for 2 hours at room temperature. Following this, the samples were dehydrated in increasing concentrations of ethanol (25%, 50%, 75%, 95% to 100% ethanol, with 95% and 100% concentrations being performed twice). The samples were allowed to air dry and mounted on stubs secured by carbon adhesive tapes. They were then sputter coated with a 10-nm-thick layer of gold (Bal-Tec) and examined a JSM-5600 scanning electron microscope (JEOL, Tokyo, Japan). The edges of the extracted capsules were then evaluated using scanning electron microscopy (SEM). The sample processing was performed as described previously [7]. For each lenticule, four micrographs, 1 in each quadrant, were taken (×140, ×750 and ×4000 magnification).

Lens capsulotomy strength was determined by an Instron 3343 mechanical tester (Instron Corp, Canton MA) after removal of the lens *en bloc* with nucleus intact or following expression with an ophthalmic viscoelastic device (Viscoat, Alcon). A customized test fixture with two mushroom shaped pins were placed posterior to the capsulotomy edge and the rate of pin displacement was set at 6 mm/min. Resistance of the capsulotomy to rupture was measured in mN and the stretching ratio by (capsulotomy size mm + displacement mm)/capsulotomy size mm.

Statistical analysis was with the Mann-Whitney U test for unpaired comparisons, the Wilcoxon matched-pairs signed rank test for paired analysis or the Kruskal-Wallis test with Dunn’s post hoc test for groups with Prism 5.0 for Macintosh.

## Additional Information

**How to cite this article**: Williams, G. P. *et al.* The effects of a low-energy, high frequency liquid optic interface femtosecond laser system on lens capsulotomy. *Sci. Rep.* **6**, 24352; doi: 10.1038/srep24352 (2016).

## Figures and Tables

**Table 1:** Summary of lens capsule strength with different femtosecond laser platforms.

FLACS Platform	Methods	Outcome
Catalys^16^	Porcine, intact lens	152 mN vs. 66 mN with manual capsulotomy
Catalys^12^	Porcine 4.6 mm diameter, intact lens	115–152 mN vs. 65 mN with manual capsulotomy
LensAR (Frey *et al.* in)^23^	Porcine 5 mm diameter, intact lens	177 mN vs. 125 mN with manual capsulotomy
LensAR^10^	Porcine 5 mm diameter, intact lens	174 mN vs. 119 mN with manual capsulotomy
Victus^15^	Porcine 5.5 mm diameter cut	113 mN vs. 73 mN with manual capsulotomy
LenSX^22^	Porcine 5 mm diameter, lens cut	119 mN vs. 155 mN with manual capsulotomy
